# Metagenomics in the Diagnosis of Pneumonia: Protocol for a Systematic Review

**DOI:** 10.2196/57334

**Published:** 2024-09-18

**Authors:** Samuel Quarton, Alana Livesey, Charlotte Jeff, Christopher Hatton, Aaron Scott, Dhruv Parekh, David Thickett, Alan McNally, Elizabeth Sapey

**Affiliations:** 1 National Institute for Health Research Birmingham Biomedical Research Centre Birmingham United Kingdom; 2 Institute of Inflammation and Ageing University of Birmingham Birmingham United Kingdom; 3 National Institute for Health Research Welcome Trust Clinical Research Facility University Hospitals Birmingham Birmingham United Kingdom; 4 National Institute for Health Research Midlands Patient Safety Research Collaboration University of Birmingham Birmingham United Kingdom; 5 National Institute for Health Research Midlands Applied Research Collaborative University of Birmingham Birmingham United Kingdom

**Keywords:** pneumonia, metagenomics, CAP, community-acquired pneumonia, HAP, hospital-acquired pneumonia, VAP, ventilator-associated pneumonia, diagnosis, respiratory tract infection, systematic review

## Abstract

**Background:**

Causative pathogens are currently identified in only a minority of pneumonia cases, which affects antimicrobial stewardship. Metagenomic next-generation sequencing (mNGS) has potential to enhance pathogen detection due to its sensitivity and broad applicability. However, while studies have shown improved sensitivity compared with conventional microbiological methods for pneumonia diagnosis, it remains unclear whether this can translate into clinical benefit. Most existing studies focus on patients who are ventilated, readily allowing for analysis of bronchoalveolar lavage fluid (BALF). The impact of sample type on the use of metagenomic analysis remains poorly defined. Similarly, previous studies rarely differentiate between the types of pneumonia involved—community-acquired pneumonia (CAP), hospital-acquired pneumonia (HAP), or ventilator-associated pneumonia (VAP)—which have different clinical profiles.

**Objective:**

This study aims to determine the clinical use of mNGS in CAP, HAP, and VAP, compared with traditional microbiological methods.

**Methods:**

We aim to review all studies (excluding case reports of a series of fewer than 10 people) of adult patients with suspected or confirmed pneumonia that compare metagenomic analysis with traditional microbiology techniques, including culture, antigen-based testing, and polymerase chain reaction–based assays. Relevant studies will be identified through systematic searches of the Embase, MEDLINE, Scopus, and Cochrane CENTRAL databases. Screening of titles, abstracts, and subsequent review of eligible full texts will be done by 2 separate reviewers (SQ and 1 of AL, CJ, or CH), with a third clinician (ES) providing adjudication in case of disagreement. Our focus is on the clinical use of metagenomics for patients with CAP, HAP, and VAP. Data extracted will focus on clinically important outcomes—pathogen positivity rate, laboratory turnaround time, impact on clinical decision-making, length of stay, and 30-day mortality. Subgroup analyses will be performed based on the type of pneumonia (CAP, HAP, or VAP) and sample type used. The risk of bias will be assessed using the QUADAS-2 tool for diagnostic accuracy studies. Outcome data will be combined in a random-effects meta-analysis, and where this is not possible, a narrative synthesis will be undertaken.

**Results:**

The searches were completed with the assistance of a medical librarian on January 13, 2024, returning 5750 records. Screening and data extraction are anticipated to be completed by September 2024.

**Conclusions:**

Despite significant promise, the impact of metagenomic analysis on clinical pathways remains unclear. Furthermore, it is unclear whether the use of this technique will alter depending on whether the pneumonia is a CAP, HAP, or VAP or the sample type that is collected. This systematic review will assess the current evidence base to support the benefit of clinical outcomes for metagenomic analysis, depending on the setting of pneumonia diagnosis or specimen type used. It will identify areas where further research is needed to advance this methodology into routine care.

**Trial Registration:**

PROSPERO CRD42023488096; https://tinyurl.com/3suy7cma

**International Registered Report Identifier (IRRID):**

DERR1-10.2196/57334

## Introduction

### Background

Pneumonia is an acute infection of lung parenchyma [1]; it is of key importance globally with high incidence and mortality [2]. Lower respiratory tract infections, encompassing pneumonia, are the leading infectious cause of death worldwide [3], although figures for incidence and mortality rates vary significantly with the population being studied. In adults, pneumonia is predominantly a disease of aging, with incidence rising from 1.1 to 4.8 per 1000 persons per year in all adults to 6.7 to 42 per 1000 persons per year in those older than 65 years old [2]. At extreme age, this increases further: a UK analysis found the rate of pneumonia episodes among 85- to 89-year-olds was 7 times that among 65 to 69-year-olds [4]. With populations aging in many countries, there is an urgent need to improve outcomes for patients with pneumonia.

The diagnosis of pneumonia is not pathogen specific, encompassing a range of infections by varied bacterial, viral, and fungal causes [5]. Prognoses and optimal treatments vary depending on the causative organism, and the likely pathogens change depending on the setting in which infection occurs, with pneumonia usually categorized into community-acquired pneumonia (CAP), hospital-acquired pneumonia (HAP), or ventilator-associated pneumonia (VAP). Identifying the responsible pathogen carries importance in allowing targeted effective treatment. However, with existing microbiological methods, an organism is only identified in around half of cases of CAP [6] and between 4% and 27% of patients with HAP [7-9]. In addition, traditional culture-based techniques have a relatively slow turnaround time of 2-5 days from sample collection to antimicrobial sensitivity results [10]. As a result, treatment is largely empirical, based on common organisms and resistance patterns, with the risk of antibiotic prescriptions that are unnecessary, ineffective, or harmful [11]. Empiric treatment, especially with broad-spectrum antibiotics as recommended for HAP and VAP [12], also drives antimicrobial resistance, which is a growing societal concern with an estimated 4.95 million deaths associated with antibiotic resistance in 2019 [13]. With lower respiratory tract infections being the largest contributor to this total [13], antibiotic resistance is both a consequence and cause of inappropriate antibiotics in pneumonia. Timely identification of causative organisms and potential resistance patterns could lead to early rationalization or escalation of antibiotics, to improve outcomes and limit the impact on antimicrobial resistance.

Metagenomic next-generation sequencing (mNGS) is an emerging technology that is of considerable promise in pneumonia [14]. Current techniques to identify a causative pathogen in pneumonia rely largely on microscopy and culture. These traditional methods have significant limitations which contribute to the low rates of pathogen identification. Not all pathogens grow readily in standard culture media, and where organisms are identified results often take several days to be reported, leading to delays in optimizing antimicrobials [10,15]. Metagenomic sequencing offers a nonbiased approach to pathogen identification by sequencing all nucleic acid present in a sample and so, in addition to identifying organisms, has the potential to provide data on likely resistance patterns or virulence. It also offers potential improvement in laboratory turnaround times, with 24 hours having been achieved in clinical practice [16], with a 6-hour turnaround from sample to result in a research environment, suggesting what may become possible with scale and optimization [17].

Increasingly, studies are considering the role of metagenomics in pneumonia diagnosis [18-20]. This has led to a systematic review of the sensitivity and specificity of metagenomics for pneumonia diagnosis [21]. However, work assessing the clinical use of metagenomics is more limited. Lv et al [22] performed a recent systematic review assessing relative pathogen detection rates, with some clinical outcomes; however, this only considered severe pneumonia with the majority of included studies based exclusively within intensive care. Similarly, a recent pilot of metagenomics within a clinical service showed a significant impact on antimicrobial prescribing but was again based solely on intensive care [14]. Most patients with pneumonia are not treated in the intensive care unit, and it is important to assess the potential benefit of metagenomic methods outside this setting. In addition, the Lv et al [22] review also made no distinction between CAP, HAP, or VAP. Given that CAP, HAP, and VAP result from a different range of pathogens, among differing cohorts of patients, it is possible that metagenomics may have clinical use in one disease but be ineffective in another.

Studies have predominantly looked at the impact of metagenomic analysis of bronchoalveolar lavage samples, and the relative sensitivity and specificity of other bodily fluids (such as expectorated sputum, pharyngeal swabs, or peripheral blood sampling) remains uncertain for patients with pneumonia. Lower respiratory tract sampling may improve pathogen identification but is impractical to obtain for many patients. Understanding the relative use of less invasive sampling methods is important.

While previous work has looked specifically at the sensitivity and specificity of metagenomics, the clinical relevance of this methodology remains unclear. To be routinely adopted, metagenomics will need to identify a causative pathogen from nonsterile samples, improve the timeliness of laboratory turnaround times, impact clinical prescribing, and be associated with improvements in clinical outcomes. These may differ in CAP, HAP, and VAP.

### Objectives

This systematic review aims to determine the clinical use of mNGS in CAP, HAP, and VAP among an adult population, compared with traditional microbiological methods. This will be through assessing the impact of mNGS on the frequency and speed of pathogen identification, as well as evidence of this translating to clinical outcomes, such as change in antibiotic therapy. It will also include subgroup analyses on how the use of mNGS is affected by the sample type used for metagenomic analysis.

## Methods

The review will be conducted and reported in keeping with PRISMA (Preferred Reporting Items for Systematic reviews and Meta-Analyses) guidelines [23], and a completed PRISMA checklist will accompany any published results.

### Eligibility Criteria

Studies comparing metagenomic analysis for identifying pathogens with traditional microbiological methods (to include culture, serum, and urine antigen testing and polymerase chain reaction–based approaches) will be included (Textbox 1). As clinical use of metagenomic analysis is an emerging field, case series and observational studies will be included, as well as randomized controlled trials. No date or language restrictions will be applied. The primary and secondary outcomes included in the systematic review are shown in Textbox 2.

**The inclusion and exclusion criteria.**

**Inclusion criteria**

- Randomized controlled trials, observational studies, and case series of more than 10 patients
- Studies comparing metagenomic analysis with alternative methods of microbiological diagnosis
- Studies report data for hospitalized patients with suspected pneumonia (community-acquired pneumonia, hospital-acquired pneumonia, or ventilator-associated pneumonia)
- Studies that include adult patients only (aged 18 years old and older)

**Exclusion criteria**

- Case series of less than 10 patients
- Studies describe respiratory infection, but where the definition does not require any radiological changes in keeping with pneumonia
- Animal or environmental studies

**Primary and secondary outcomes.**

**Primary Outcome**

- Pathogen positivity rate of metagenomic analysis in confirmed community-acquired pneumonia, hospital-acquired pneumonia, and ventilator-associated pneumonia compared with standard methods (to include culture, antigen testing, and polymerase chain reaction)

**Secondary Outcomes**

- The primary outcome analyzed in subgroups based on sample type (blood, sputum, or bronchoalveolar lavage fluid
- Laboratory turnaround time (defined as the time from sample receipt to report of organisms identified, with antimicrobial sensitivities where available)
- Impact on clinical decision-making (to include proportion of patients in which antimicrobials were changed or rationalized based on metagenomic results)
- Length of stay (hospital admission)
- 30-day and 90-day mortality
- Sensitivity and specificity of metagenomic methods for diagnosing community-acquired pneumonia, hospital-acquired pneumonia, and ventilator-associated pneumonia

### Search Strategy

A comprehensive search of Embase, MEDLINE (through the PubMed interface), Scopus, and Cochrane CENTRAL databases will be performed, with the support of a health care librarian, as well as gray literature. Reference lists will also be manually searched for appropriate studies. No date or language restrictions will be applied.

### Search Terms

Exact search strings will be optimized for the database being searched. The full search strategy used will be provided as supplementary material with the completed review.

The following provides the core terms around which searches will be based: (pneumonia OR CAP OR HAP OR VAP OR “lung infection” OR “respiratory infection” OR “pulmonary infection”) AND (metagenomic* OR “next generation sequencing” OR “NGS” OR “mNGS”).

### Screening, Data Management, and Data Extraction

Studies identified by searches will be stored and processed using the “Covidence” software program package (Covidence). Retrieved titles and abstracts will be independently assessed for eligibility by 2 reviewers (SQ and 1 of AL, CJ, or CH), with a third independent clinician (ES) providing adjudication in cases of disagreement. Full text of eligible articles will be retrieved, and data relevant to our primary and secondary outcomes will be extracted and recorded onto predesigned forms. Where studies that provide separate data for CAP, HAP, or VAP, or explicitly study only 1 of these, this will be recorded and combined outcomes will be reported for each pneumonia type. If the type of pneumonia is not clear or the criteria used to define pneumonia do not allow separation into subtypes, data will still be collected to avoid the loss of meaningful information. These data will only be included in outcomes looking at the use of metagenomics in pneumonia as a whole. Where reported, data relevant to diagnostic test accuracy (true positive, false positive, true negative, and false negative) will be recorded to enable pooled sensitivity and specificity calculations. Information will also be extracted regarding study design, including sample size, definitions of pneumonia used methods for randomization where appropriate, and the criteria used for a reference standard of positive diagnosis, as well as declared funding sources.

For papers where the sample type used for analysis is not stated or only statistics of combined sample types are given, attempts will be made to contact the authors to retrieve this missing information. If this is not available, these data will not be used when analyzing subgroups by sample type but will still be included for other outcomes. Other missing data will be recorded and reported on when discussing results.

As this is a systematic review of a diagnostic tool, the quality of included studies and risk of bias will be assessed using the QUADAS-2 tool (University of Bristol), as determined by 2 independent reviewers (SQ, AL, CJ, or CH). Again, any disagreement during data extraction or in the assessment of study quality will be discussed with a third reviewer (ES) and a consensus decision reached.

### Analysis of Results

Statistical analyses will be conducted using R version 4.2 (The R Collaboration). For outcomes where insufficient data are available to conduct a meta-analysis, this will be highlighted, and the data will be combined in a narrative synthesis.

### Reporting of Outcome Measures

Data from eligible studies will be pooled, and if possible, random effects meta-analyses of outcome measures will be performed. For dichotomous outcomes, we will calculate odds ratios with 95% CIs where possible. Laboratory turnaround times, as a continuous outcome, will be converted to standard units (hours) and the mean difference will be calculated. Subgroup analyses will be performed to look at the type of sample used for metagenomic analysis, and analysis will also be performed separately for CAP, HAP, and VAP.

### Missing Data

Where SDs are not provided for continuous outcome data (such as laboratory turnaround time), study authors will be contacted up to 3 times in an attempt to obtain these. If this is not possible, attempts will be made to estimate SDs using recognized formulae from statistics provided (eg, standard error or *P* values), or if there is insufficient information to allow this, an SD value will be imputed based on that of other included studies. Where this is necessary, a sensitivity analysis of the results will be performed to identify if this has a meaningful impact.

### Assessing Heterogeneity

A funnel plot will be performed to assess for publication bias, and statistical heterogeneity of studies will be calculated using *I^2^*. Where substantial heterogeneity is found (*I^2^*>50%), data entry will be assessed for accuracy. Possible reasons for the observed heterogeneity will be investigated by identifying studies that are obvious outliers on visual inspection of the graphical data and assessing for any methodological or population characteristics that account for the heterogeneity. These will be discussed alongside the results.

## Results

Searches were completed on January 13, 2024, returning 5750 records. Screening of returned papers and data extraction are anticipated to be completed by September 2024. The review is registered prospectively on the PROSPERO database (CRD42023488096) and will be updated once complete. The results will be submitted for publication in peer-reviewed journals and presentation at local and international conferences.

## Discussion

A systematic review of the literature on the use of metagenomics in the management of pneumonia should clarify the current understanding of this topic of importance in terms of clinical burden and current existing unmet needs. Metagenomic sequencing has been shown previously to improve the rate of pathogen identification [21]. However, how to interpret this in light of a nonsterile respiratory tract and lack of gold-standard comparators is challenging. The clinical significance of this increased analytical sensitivity remains unclear. Assessing the impact on clinical outcomes will highlight the potential clinical relevance or otherwise of metagenomics in this context and highlight where future studies may be needed to develop this further. Similarly, it will clarify if mNGS may be of particular benefit in certain pneumonia subtypes, and by investigating the impact of different sample types, it may identify the least invasive method to provide meaningful clinical data, given that existing reviews have focused on the use of BALF [21]. If insufficient data currently exist to make clear conclusions, this will be highlighted by the review and so direct where future research is needed.

We feel these are questions of clinical importance, and this review will offer a valuable contribution to shaping the use of emerging technologies in clinical practice. The results will be submitted for publication in peer-reviewed journals and presented at both local and international conferences. If, for any outcome, we are unable to identify sufficient studies to derive meaningful results, this will be reported to help direct future avenues for research.

## Abbreviations

BALF: bronchoalveolar lavage fluid
CAP: community-acquired pneumonia
HAP: hospital-acquired pneumonia
mNGS: metagenomic next-generation sequencing
PRISMA: Preferred Reporting Items for Systematic reviews and Meta-Analyses
VAP: ventilator-associated pneumonia

## Data Availability

Data sharing is not applicable to this article as no datasets were generated or analyzed. On completion of the study, datasets generated as part of the study will be included in any submissions for publication.
